# Validity and reliability of local endemic language version of the SARI Sigma Scale questionnaire for assessing stigma in leprosy patients

**DOI:** 10.3389/fpubh.2024.1474745

**Published:** 2025-01-08

**Authors:** Tri Rahayu, Dewi Friska, Yoslien Sopamena, Sri Linuwih Menaldi, Kahlil Gibran, Dadun, Ida Ruwaida, Yunia Irawati

**Affiliations:** ^1^Department of Ophthalmology, Faculty of Medicine, Universitas Indonesia—Dr. Cipto Mangunkusumo Hospital, Jakarta, Indonesia; ^2^Department of Community Medicine, Faculty of Medicine, Universitas Indonesia—Dr. Cipto Mangunkusumo, Jakarta, Indonesia; ^3^Department of Health Education and Behavioral Sciences, Faculty of Public Health, Universitas Indonesia, Jakarta, Indonesia; ^4^Department of Dermatology and Venereology, Faculty of Medicine, Universitas Indonesia—Dr. Cipto Mangunkusumo Hospital, Jakarta, Indonesia; ^5^Faculty of Medicine, Universitas Indonesia—Dr. Cipto Mangunkusumo, Jakarta, Indonesia; ^6^Center for Health Research, Faculty of Public Health, Universitas Indonesia, Jakarta, Indonesia; ^7^Faculty of Social and Political Sciences, Universitas Indonesia, Jakarta, Indonesia

**Keywords:** leprosy, reliability, SARI Stigma Scale, stigma, validity

## Abstract

**Introduction:**

The Stigma Assessment and Reduction of Impact (SARI) Stigma Scale is an instrument developed to evaluate stigma in Leprosy patients. Despite existing versions in Indonesian, the absence of an endemic area language version of a reliable assessment tool presents a barrier to effective interventions in regions like Ambon. This study aims to evaluate the validity and reliability of the Ambonese-Malay Language of SARI Stigma Scale questionnaire.

**Methods:**

A cross-sectional study involved 50 participants with leprosy or a history of leprosy from Ambon City, Indonesia. They were tested with the SARI Stigma Scale questionnaire, consisting of 4 domains of questions, totalling 21 questions. Reliability and variability analysis was conducted from each domain’s questions. Cronbach’s α (CA) and intraclass correlation coefficient (ICC) determined internal consistency of reliability tests. For validity assessment, coefficients of corrected item-total correlation ensured scale accuracy for measuring stigma.

**Results:**

Reliability analysis revealed significant high internal consistency (α and ICC value >0.7) across all four domains, with CA values ranging from 0.71 to 0.94 and strong consistency among responses, with ICC ranging from 0.71 to 0.94 across domains. The total domain exhibited a CA of 0.855 and an average ICC of 0.855 (*p* < 0.001). Validity testing demonstrated significant moderate to strong correlations, ranging from 0.69 to 0.90 (p < 0.001), affirming scale validity in measuring stigma accurately.

**Discussion:**

The Ambonese-Malay version of the SARI Stigma Scale exhibits validity and reliability as an assessment tool for scoring stigma in leprosy patients in Ambon. Stigma can emerge and be associated with leprosy. To understand the stigma in society due to this disease, a validated questionnaire in the local language and adjusted with the local cultures needed.

## Introduction

Leprosy, caused by *Mycobacterium leprae*, results in severe skin lesions, nerve damage, and disability if untreated. Early detection is crucial to prevent complications (1). Stigma, however, remains a significant barrier to the effective elimination of leprosy, as it leads to discrimination and social exclusion (2). Indonesia, with its 718 local languages as of 2019, faces unique linguistic and cultural challenges, especially in regions where Indonesian is not universally understood (3). Maluku Province, home to 62 languages and 1,390 islands, reported a significant leprosy burden, with 360 cases in 2022 (4–6). Ambon, the capital of Maluku, reported 131 cases between 2018 and 2022, highlighting the persistent challenges of addressing stigma in treating leprosy, particularly in eastern Indonesia (6–8).

Stigma refers to negative social attitudes and discrimination against individuals based on perceived undesirable traits, leading to social exclusion (9). In leprosy, stigma is particularly harmful due to misconceptions about its contagiousness, fueled by fear and superstition. This results in the marginalization of individuals with leprosy, as well as their families and communities. The visible deformities caused by the disease often lead to social isolation, making it difficult for affected individuals to participate in everyday activities, including work, education, and religious practices. This exclusion exacerbates emotional and psychological burdens, sometimes leading to depression or suicidal thoughts (10).

Additionally, stigma creates barriers to effective treatment, causing delays in diagnosis, treatment non-compliance, and increased transmission, ultimately worsening the disease’s impact. The physical disabilities associated with leprosy, such as deformities in the hands, feet, and eyes, further limit personal and professional opportunities, reducing overall quality of life (11).

The SARI Stigma Scale has proven effective in assessing stigma experienced by leprosy patients. Originally validated in Indonesian, the SARI scale provides an important framework for understanding and mitigating stigma’s impact (12, 13). In Sri Lanka, for example, the SARI scale was translated into Sinhalese, validated using confirmatory factor analysis (CFA), and demonstrated adequate internal consistency (Cronbach’s alpha = 0.74) and reproducibility. The study confirmed the scale’s effectiveness in assessing stigma across cultural contexts while retaining its core four-factor structure (experienced, disclosure, internalized, and anticipated stigma) (14).

Until now, leprosy remains a major global health issue, with over 200,000 new cases reported annually in more than 120 countries (15). In 2023, Indonesia reported 14,376 new cases, with a prevalence rate of 62.2 per 1,000,000 population (16, 17). As of January 2024, Maluku reported 301 cases, including 13 paucibacillary (PB) and 288 multibacillary (MB) cases, making it one of seven provinces yet to achieve Indonesia’s national leprosy elimination target (18, 19).

Based on these findings, our study aims to validate the SARI scale in the Ambonese-Malay language, ensuring its cultural and linguistic applicability for addressing stigma in Ambon and other leprosy-endemic regions in Indonesia (20).

## Materials and methods

### Study design and procedure

A cross-sectional study was conducted in Ambon City, Indonesia, in July 2022, involving 50 participants diagnosed with leprosy or with a history of the disease. The study was carried out by a community health service initiative programme known as *Identifikasi Tanda-Tanda Mata, Ekstremitas, dan Kulit pada Kusta Universitas Indonesia* (KATAMATAKU UI).

### Participants

The inclusion criteria comprised individuals who had completed elementary school and were over 13 years old, with a confirmed diagnosis of leprosy or a documented history of the disease.

### Data collection

The data was obtained through a structured interview with the participant by reading the question items in modified local language and dialect to ensure the participant understood the 21 question items in question. For participants under the age of 18, parental consent was obtained for their participation in the interview. The interviews were carried out by three researchers from the fields of public health, medicine, and social sciences who had received prior training.

### The SARI stigma scale

The study utilized the SARI Stigma Scale, a validated Indonesian-language instrument established in 2017. Specifically crafted to assess and alleviate the stigma surrounding leprosy, this scale comprises four domains: Experienced stigma, Disclosure concerns, Internalised stigma, and Anticipated stigma. Each domain aims to capture different facets of the stigma experienced by individuals affected by leprosy. With a total of 21 questions, the SARI Stigma Scale explores how patients encounter stigma in their daily lives. The scoring method used is that if the patient answers no, do not know, or it is not relevant, they will be given a score of zero, whereas if they answer yes, the patient will receive a score ranging from one to three depending on the frequency the patient experiences. Regarding the questionnaire, please refer to the Appendix. The availability and applicability of this instrument made it a suitable choice for our study (12).

### Translation process

The SARI Stigma Scale was previously available in an Indonesian language version, which was adapted. In order to be applicable to community groups in Ambon using the Ambon-Malay dialect, it was preceded by an adaptation process. The iterative translation process into Ambonese-Malay language was guided by the International Society of Pharmacoeconomics and Outcomes Research (ISPOR) (21).

**Figure 1:**
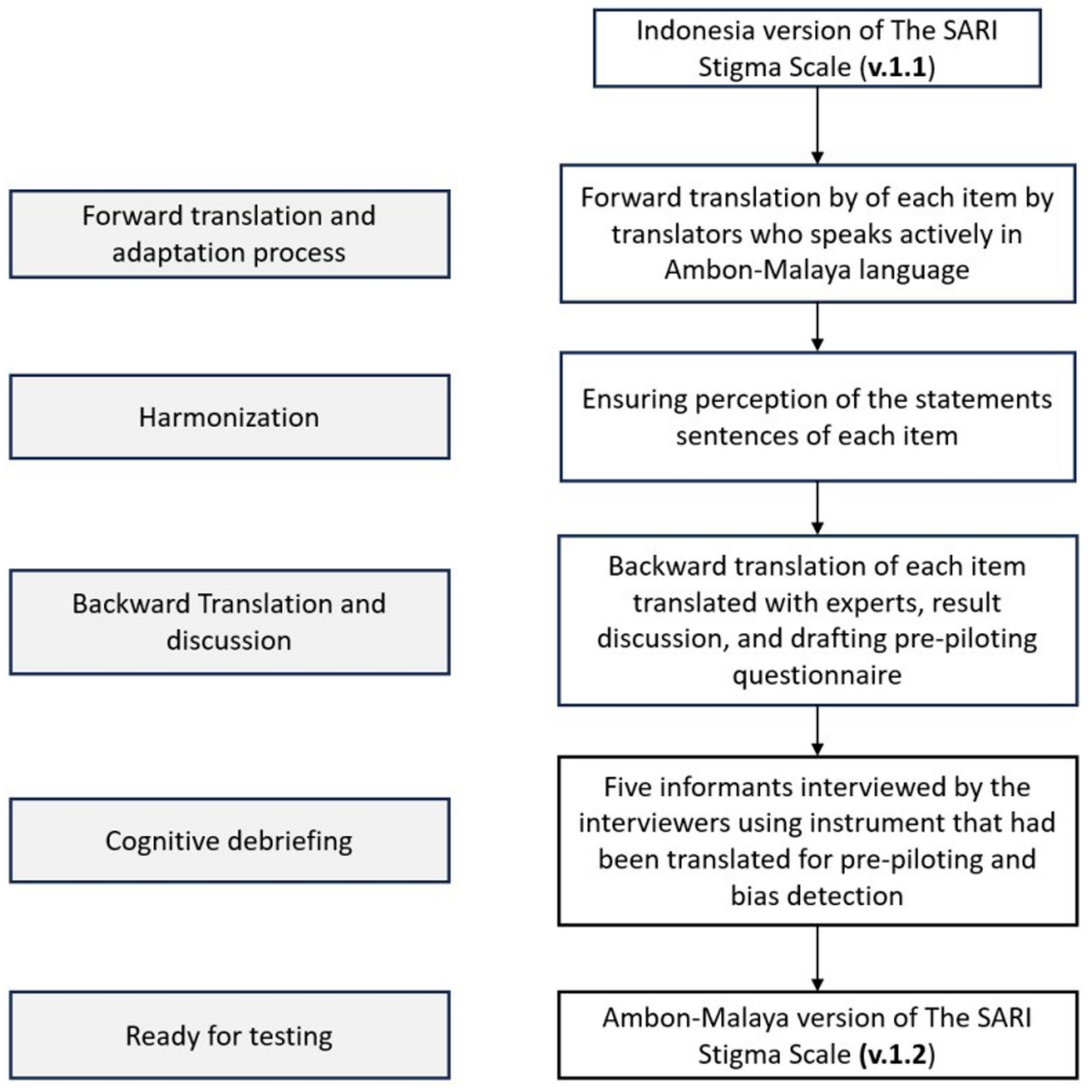
The translation process of the SARI Stigma Scale language into the Ambonese-Malay Version following the ISPOR guidelines.

The translation and adaptation process of the SARI Stigma Scale involved several steps to create the Ambon-Malaya version (v.1.2) from the Indonesian version (v.1.1). The process began with forward translation by translators fluent in the Ambon-Malay language, followed by ensuring the perception of the statements in each item was maintained. Next, a backward translation was performed by experts, including discussions and drafting of a pre-piloting questionnaire. Afterward, cognitive debriefing was conducted, during which five informants were interviewed by interviewers using the translated instrument to identify potential biases. Once this process was complete, the Ambon-Malay version of the scale was deemed ready for testing (Figure 1).

The discussion of each item of the SARI Stigma Scale questionnaire was conducted by a team led by researchers who carried out the validation and adaptation process of the SARI Stigma Scale Indonesian language format, consisting of researchers from the University of Indonesia, the KATAMATAKU Team, researchers from Pattimura University, Maluku, and sociologists from the Indonesian Christian University of Maluku. All of these processes took place at Pattimura University, Ambon, Indonesia.

Compared to the original version of the Sari Stigma Scale, which has scores ranging from one to three depending on the frequency of “yes” responses from patients, the Ambonese-Malay version of the Sari Stigma Scale simplifies the scoring to only one and two. This simplification is done because there is no reference or defined time frame available for distinguishing between “Always/Often,” “Sometimes,” and “Rarely/once” to convey to participants. Additionally, this is also to facilitate ease of answering the questionnaire for participants who may be considered less familiar with it.

The discussion involved reading each statement item and translating it into the Ambonese-Malay dialect. After the translation process, the items were read aloud together to ensure there were no differences in perception of the statement sentences. To ensure that the substance of each item remained consistent with the original Indonesian language version of the SARI Stigma Scale, discussions were held again with experts who had conducted the adaptation process.

Five participants were then invited to conduct pre-piloting through interviews conducted by the interviewers. The results of these five interviews were then discussed with the research team to see if there were any biases in the instrument that had been translated. No changes were made, indicating that the instrument, which had undergone language translation, was ready for testing.

### Statistical analysis

Demographic and clinical characteristics of the study population were included. Two measures of descriptive statistics include the mean and standard deviation (SD) of raw responses. Data was recorded in software Microsoft Excel and analysed using statistical analysis software.

### Validity and reliability test

Item analysis using corrected item-total correlation coefficients was performed for questions one to 21 to assess the relationship between individual items and the total score of their respective domains. This method ensures that each item is consistent with the construct it aims to measure. The validation conducted in this study was limited to internal validation. A question item was considered valid if the corrected item-total correlation value was greater than 0.30.

The internal consistency of the SARI Stigma Scale questionnaire was evaluated using Cronbach’s α (CA). CA values were interpreted as above 0.7 indicating good internal consistency. Additionally, the intraclass correlation coefficient (ICC) was calculated to determine the stability of responses across domains. ICC values were interpreted as 0.7 indicating good stability. These analyses were conducted for each of the four domains separately and for the total domain to ensure the reliability of the instrument in measuring stigma among participants.

### Ethical aspects

The study received approval from the Ethics Committee of the Faculty of Medicine, Universitas Indonesia (KET-733/UN2.FI/ETIK/PPM.00.02/2022, date of approval: 25 July 2022). Informed consent was obtained from all participants. The study adhered to the principles outlined in the Helsinki Declaration.

## Results

### Cognitive debriefing notes

During the cognitive debriefing sessions conducted by a local enumerator with five participants, it became apparent that several sentence structures were adapted from Malay language with the Ambon dialect. Additionally, everyday expressions and words commonly used in the local context were also observed. There were no specific sentence translations that were of concern and significantly affected the questionnaire.

### Participant characteristics

A total of 50 participants were involved in this study, all of whom were eligible and completed answering all 21 questions from the interview. Additionally, all participants provided complete characteristic data. Table 1 shows the baseline characteristics of the study population. The majority of participants were male (78%), with the highest proportion aged between 16 to 25 years (40%). Most participants had completed senior high school (65%) and were either unemployed (38%) or private-sector employees (32%). In terms of districts, Nusaniwe had the highest representation (34%). Regarding marital status, the majority were single (54%), and 90% of participants showed signs of leprosy. Monthly income varied, with 60% earning less than Rp2,860,000, the minimum wage in Ambon City for 2023.

**Table 1:** Participant characteristics.

Characteristics (*n* = 50), *n* (%)			
Age (years)		District in Ambon City	
13–15	2 (4%)	Sirimau	11 (22%)
16–25	20 (40%)	Nusaniwe	17 (34%)
26–35	8 (16%)	Teluk Ambon	5 (10%)
36–45	5 (10%)	Lei Timur Selatan	3 (6%)
>46	15 (30%)	Baguala	12 (24%)
Sex (male/female)		Leihitu	2 (4%)
Male	39 (78%)	Marital status	
Female	11 (22%)	Single (Never married)	27 (54%)
Education		Married	21 (42%)
Elementary School graduate	3 (6%)	Divorced (living/deceased)	2 (4%)
Junior High School graduate	6 (12%)	Monthly income*	
Senior High School graduate	34 (65%)	> = Rp2.860.000	20 (40%)
University graduate	7 (14%)	<Rp2.860.000	30 (60%)
Occupation		Sign of leprosy	
College Students	6 (12%)	Yes	45 (90%)
Laborer/Worker	6 (12%)	No	5 (5%)
Civil servant	3 (6%)		
Private-sector employees	16 (32%)		
Unemployed	19 (38%)		

This includes demographic details, domicile, and signs of leprosy, based on data collected in Ambon City (*n* = 50). (*) The minimum wage in Ambon City for 2023 is Rp2,860,000.

### Validity analysis

The relationship between each individual item on a scale using corrected item-total correlation shown in Tables 2, 3. For experienced stigma, all items have strong correlations with the total score, ranging from 0.69 to 0.90, indicating that these items are well-aligned with the overall construct being measured by the scale. For disclosure concerns, the correlations range from 0.45 to 0.62. For internalized stigma, the correlations range from 0.36 to 0.61. For questions anticipated stigma, the correlations range from 0.42 to 0.59. These items show a mix of moderate to relatively strong correlations with the total score, indicating their varying degrees of alignment with the construct being measured. Question number 14 and 16 has the lowest score for all questions (0.35 and 0.36).

**Table 2:** Variability and reliability for all 21 questions of Ambonese-Malay language version of the SARI Stigma Scale questionnaire translated.

Questions		Corrected Item-Total Correlation	Cronbach’s Alpha	Intraclass Correlation Coefficient
Total all the four domains (items = 21)			0.855	0.855
Domain 1. Experienced Stigma (items = 7)			0.94	0.94 (0.92–0.96)
1	Do some people who know that you have (had) leprosy keep more distance from you?	0.69		
2	Do people you care about stop contacting you after learning you have (had) leprosy?	0.73		
3	Did you lose friends by telling them you have(had) leprosy?	0.87		
4	Do people avoid touching you after knowing that you have(had) leprosy?	0.86		
5	Have people physically backed away from you when they learn that you have(had) leprosy?	0.78		
6	Do people seem afraid of you once they learn you have(had) leprosy?	0.90		
7	Do you feel set apart and isolated from the community since learning you have(had) leprosy?	0.80		
Domain 2. Disclosure concerns (items = 4)			0.73	0.73 (0.58–0.83)
8	Are you careful who you tell that you have (had) leprosy?	0.45		
9	Do you feel the need to hide your leprosy?	0.59		
10	Do you believe telling someone you have (had) leprosy is risky?	0.62		
11	Do you worry that people may judge you when they hear you have(had) leprosy?	0.45		

This includes item-total correlations, Cronbach’s alpha, and intraclass correlation coefficients for the total scale and its domains: Experienced Stigma and Disclosure Concerns.

**Table 3:** Variability and reliability for all 21 questions of Ambonese-Malay language version of the SARI Stigma Scale questionnaire translated.

Questions		Corrected item-total correlation	Cronbach’s alpha	Intraclass correlation coefficient
Total all the four domains (items = 21)			0.855	0.855
Domain 3. Internalized Stigma (items = 6)			0.74	0.74 (0.62–0.84)
12	Do you feel guilty because you have(had) leprosy?	0.54		
13	Do you feel you are not as good a person as others because you have (had) leprosy?	0.56		
14	Are you embarrassed that you have (had) leprosy?	0.36		
15	Does having (had) leprosy make you feel unclean?	0.61		
16	Do you regret having told some people that you have (had) leprosy?	0.35		
17	Does having(had) leprosy make you feel that you are a bad person?	0.50		
Domain 4. Anticipated Stigma (items = 4)			0.71	0.71 (0.56–0.82)
18	Do people affected by leprosy lose their jobs when their employers find out?	0.52		
19	Are people affected by leprosy treated like a public nuisance?	0.42		
20	Do most people think that a person affected by leprosy is disgusting?	0.59		
21	Do most people feel uncomfortable around someone affected by leprosy?	0.52		

This includes item-total correlations, Cronbach’s alpha, and intraclass correlation coefficients for the total scale and its domains: Internalized Stigma and Anticipated Stigma.

### Reliability analysis

The CA and ICC for each of the four domains shown in Tables 2, 3. Measure of internal consistency using Cronbach’s alpha coefficient of all items, yielded a robust value of 0.855, indicating strong agreement among the items within the scale. ICC yielded a significantly higher value of 0.855 (95% CI: 0.79–0.90), indicating strong agreement between measurements.

Experienced Stigma in the domain has the highest CA and ICC. All CA coefficients and ICCs in all domains and total items yield results with statistical significance, with *p*-values <0.001, exceeding 0.7. This indicates that the SARI Stigma Scale effectively measures the same underlying construct across its 21 questions, making it a reliable tool for assessing stigma in leprosy patients.

## Discussion

### Key results

The findings of this study highlight the validity and reliability of the Ambonese-Malay language version of the SARI Stigma Scale as an effective tool for assessing stigma among leprosy patients in Ambon City, Indonesia. The reliability analysis revealed strong internal consistency across all four domains of the scale, with CA values indicating acceptable to excellent consistency (α = 0.855). Additionally, the ICC demonstrated strong stability of responses across all domains (ICC = 0.855), further supporting the reliability of the instrument.

In terms of validity, the scale exhibited all coefficients of corrected item-total correlation of each item >0.3 with significant results in measuring stigma accurately in the target population. The correlations between individual items and the total score within each domain varied, indicating the varying degrees of alignment with the overall construct of stigma.

The low scores observed for question numbers 14 and 16 (coefficients 0.35 and 0.36) in the corrected item-total correlation analysis may indicate several underlying factors contributing to their poor alignment with the overall construct being measured by the SARI Stigma Scale. Internalized stigma, which refers to the acceptance and internalization of negative beliefs, attitudes, and stereotypes associated with one’s condition, may be a significant factor influencing the small value of the corrected item-total correlation analysis for these questions. It’s possible that these questions address aspects of stigma that are particularly sensitive or taboo for participants, leading them to be less willing to express their true answers. Additionally, cultural or normative aspects within the Ambonese-Malay-speaking population may influence participants’ responses to these specific questions. It is notable that patients may exhibit a tendency to either not admit or deny certain aspects related to their condition (22).

### Strength

The translation study of the SARI Stigma Scale from common Indonesian language into local languages in Indonesia renders this research a profound examination, delving deeper into the utilization of the questionnaire. This aspect strengthens the study by enhancing its cultural relevance and ensuring a more comprehensive assessment of its applicability within the local context.

A sample size of 50 participants was used to assess the internal consistency of a 21-item questionnaire through Cronbach’s alpha. While there were initial concerns about the sample size, Bonett’s (30) guidelines show that a sample size of 50 is sufficient. For 21 items, an expected Cronbach’s alpha of 0.7, and a power of 0.90, the minimum required sample size is approximately 18. Therefore, the sample size of 50 exceeds the minimum requirement, ensuring the reliability and validity of the results (23).

### Interpretation and implication

Overall, the validated Ambonese-Malay version of the SARI Stigma Scale, alongside the existing Indonesian versions, holds promise for advancing our understanding of stigma in leprosy patients and improving the quality of care and support provided to this vulnerable population. By including participants under 18 years old in stigma assessment studies, this tool also holds promise for advancing our understanding of stigma not only among adult leprosy patients but also among pediatric populations.

Originally developed in the Indonesian language, The SARI Stigma Scale underwent a cross-cultural validation process in Cirebon District, Indonesia, initiated by the Stigma Assessment and Reduction of Impact (SARI) project and led by Dadun et al. in 2017. The Berger Scale, intended for HIV/AIDS-related stigma assessment, was adapted for evaluating leprosy-related stigma (12, 24). The validation process, including qualitative studies, translation and back-translation, interviewer training, pilot testing, and main data collection, aimed to ensure conceptual, item, semantic, operational, and measurement equivalence. Adjustments were made to address insufficient equivalences, resulting in a revised scale with improved validity and reliability. The revised scale demonstrated good internal consistency (Cronbach’s alpha 0.88), no floor or ceiling effects, and good reliability (intra-class correlation coefficient 0.75), indicating its suitability for assessing leprosy-related stigma (12).

The SARI Stigma Scale has gained attention beyond its country of origin, extending to regions like Iran. A study aimed at addressing the absence of a valid Persian version of this tool in Iran sought to translate and examine its psychometric properties. The research focused on determining face and content validity, employing confirmatory factor analysis (CFA) for construct validity assessment. Results indicated that the Persian version of the SARI tool demonstrated acceptable content and construct validity. Furthermore, its reliability, as evidenced by Cronbach’s alpha coefficient of 0.897 and ICC for test–retest reliability, was deemed acceptable (25).

In this study, item analysis was conducted using corrected item-total correlation coefficients to assess the relationship between individual items and the total score of their respective domains. External validation in this study was not performed due to satisfactory internal consistency results (all values >0.3), although some items had the lowest correlation values. As a recommendation, future research on the SARI Stigma Scale should aim to strengthen its construct validity through techniques such as exploratory factor analysis (EFA) or CFA (26). Furthermore, external validity using concurrent criterion validity should be assessed by comparing the scale against established external benchmarks to determine whether it accurately measures the intended construct (27).

Experienced stigma refers to the discrimination, prejudice, and negative treatment that individuals encounter due to their condition or identity. It arises from direct interactions with others or from the consequences of one’s condition being known to others. Disclosure concerns involve worries and anxieties individuals have about revealing their condition or identity to others. Internalised stigma is the acceptance and internalization of negative beliefs, attitudes, and stereotypes associated with one’s condition. Anticipated stigma involves the expectation or anticipation individuals have regarding the negative attitudes, beliefs, and behaviours others may hold towards them due to a particular aspect of their identity or condition. Specifically in the context of leprosy-related stigma, these concepts highlight the psychological impact of societal prejudices and discriminatory beliefs on individuals affected by leprosy (12).

A study in Nepal in 2018 revealed that the fear of discrimination and the need to hide the disease persists, particularly due to the visibility of deformities caused by leprosy. This stigma continues to impact crucial aspects of life such as marriage, employment, and social interaction (28).

A study conducted in Southeast Sulawesi, Indonesia in 2018 highlights the persistent influence of leprosy-related stigma on affected individuals. Rooted from perceptions of uncleanness and shame, this stigma obstructs treatment adherence and worsens the advancement of the disease. It infiltrates multiple aspects of life, impacting mobility and social interactions, thus presenting a significant obstacle to successful leprosy control personal treatment (29).

### Generalizability

The robust validity and reliability demonstrated by the Ambonese-Malay language version of the SARI Stigma Scale suggest its potential applicability in other local endemic regions where patients speak the same dialect. Additionally, there is a need to translate the SARI Stigma Scale into local languages in other endemic areas. This can be achieved through the translation stages implemented in this research wherever leprosy studies are conducted, ensuring that participants’ perceptions of each question in the questionnaire are well-received and accurately conveyed.

## Conclusion

The Ambonese-Malay language version of the SARI Stigma Scale questionnaire proves to be a valid and reliable tool for assessing stigma in leprosy patients in Ambon. With high internal consistency and significant validity results, this instrument offers a culturally adapted means to accurately evaluate stigma and guide interventions for individuals affected by leprosy in the region. Its robust methodology for validity assessment sets a precedent for future studies, especially if similar research will be carried out and applied in other endemic areas with local languages.

## Data Availability

The raw data supporting the conclusions of this article will be made available by the authors, without undue reservation.
