# Supplementary material for: Validity and reliability of local endemic language version of the SARI Sigma Scale questionnaire for assessing stigma in leprosy patients
Source: Front Public Health. 2025 Jan 8;12:1474745. doi: 10.3389/fpubh.2024.1474745 (PMC11750834; doi:10.3389/fpubh.2024.1474745)
Supplement: Supplementary file 1 [file Table_1.docx]

Supplementary Material

**Questionnaires**

**Table. The Sari Stigma Scale original version and Ambonese-Malay version**

| **SARI Stigma Scale v.1.2** | | No | Yes | Don't Know | Not Relevant | Always/ Often | Rarely/ Once | Score |
| --- | --- | --- | --- | --- | --- | --- | --- | --- |
| **Experienced stigma** | | | | | | | | |
| 1a | Do some people who know you have (had) leprosy keep more distance from you?  *Waktu orang-orang tau [..] kena kusta, apakah dong menjauhi [..]?* | 0 |  | 0 | 0 |  |  |  |
| b | (If yes) How often has this happened?  *Kalo iya, sebarapa sering akang terjadi par [..]?* |  |  |  |  | 2 | 1 |  |
| 2a | Do people you care about stop contacting you  after learning you have (had) leprosy?  *Waktu orang-orang yang [..] sayang tau kalau [..] kena kusta, apakah dong seng menghubungi [..] lai?* | 0 |  | 0 | 0 |  |  |  |
| b | (If yes) How often has this happened?  *Kalo iya, sebarapa sering akang terjadi par [..]?* |  |  |  |  | 2 | 1 |  |
| 3a | Did you lose friends by telling them you have (had) leprosy?  *Waktu [..] bilang par tamang-tamang kalo [..] kena kusta, apakah dong jadi seng mau batamang lai deng [..]?* | 0 |  | 0 | 0 |  |  |  |
| b | (If yes) How often has this happened?  *Kalo iya, sebarapa sering akang terjadi par [..]?* |  |  |  |  | 2 | 1 |  |
| 4a | Do people avoid touching you once they know you have (had) leprosy?  *Waktu orang-orang tau [..] kena kusta, apakah dong su seng mau sonto (contoh: jabat tangan) lai ?* | 0 |  | 0 | 0 |  |  |  |
| b | (If yes) How often has this happened?  *Kalo iya, sebarapa sering akang terjadi par [..]?* |  |  |  |  | 2 | 1 |  |
| 5a | Have people physically backed away from you  when they learn you have (had) leprosy?  *Waktu orang-orang tau [..] kena kusta, apakah dong su seng mau baku dekat (bikin jarak) deng [..] lai?* | 0 |  | 0 | 0 |  |  |  |
| b | (If yes) How often has this happened?  *Kalo iya, sebarapa sering akang terjadi par [..]?* |  |  |  |  | 2 | 1 |  |
| 6a | Do people seem afraid of you once they learn you have (had) leprosy?  *Waktu orang-orang tau [..] kena kusta, apakah dong kaya taku deng [..]?* | 0 |  | 0 | 0 |  |  |  |
| b | (If yes) How often has this happened?  *Kalo iya, sebarapa sering akang terjadi par [..]?* |  |  |  |  | 2 | 1 |  |
| 7a | Do you feel set apart and isolated from the community since learning you have (had) leprosy?  *Waktu [..] tau [..] kena kusta, apakah [..] rasa dijauhi deng dikucilkan dari masyarakat ?* | 0 |  | 0 | 0 |  |  |  |
| b | (If yes) How often has this happened?  *Kalo iya, sebarapa sering akang terjadi par [..]?* |  |  |  |  | 2 | 1 |  |
|  |  | ***Sub total*** | | | | | |  |

| **Disclosure concerns** | | | | | | | | | |
| --- | --- | --- | --- | --- | --- | --- | --- | --- | --- |
| 8a | Are you careful who you tell that you have (had) leprosy?  *Apakah [..]berhati-hati bacarita par orang tertentu bahwa [..]kana kusta ?* | 0 |  | 0 | 0 |  |  |  | |
| b | (If yes) How often are you careful?  *Kalo iya, sebarapa sering akang terjadi par [..]?* |  |  |  |  | 2 | 1 |  | |
| 9a | Do you feel the need to hide your leprosy?  *Apakah [..]rasa perlu seng kasi tau orang, kalo [..]kana kusta ?* | 0 |  | 0 | 0 |  |  |  | |
| b | (If yes) How often do you feel the need to hide your status?  *Kalo iya, sebarapa sering akang terjadi par [..]?* |  |  |  |  | 2 | 1 |  | |
| 10a | Do you believe telling someone you have (had) leprosy is risky?  *Apakah [..]rasa ada risikonya untuk [..]kalo carita par orang, kalo pernah atau sementara kana kusta?* | 0 |  | 0 | 0 |  |  |  | |
| b | (If yes) How often do you believe it is risky?  *Kalo iya, sebarapa sering akang terjadi par [..]?* |  |  |  |  | 2 | 1 |  | |
| 11a | Do you worry that people may judge you when  they hear you have (had) leprosy?  *Apakah [..] takut kalo orang bisa nilai [..] sebagai orang yang seng bae pas dong dengar [..] kana kusta?* | 0 |  | 0 | 0 |  |  |  | |
| b | (If yes) How often do you worry about this?  *Kalo iya, sebarapa sering akang terjadi par [..]?* |  |  |  |  | 2 | 1 |  | |
|  |  | ***Sub total*** | | | | | | |  |

| **Internalized stigma** | | | | | | | | | |
| --- | --- | --- | --- | --- | --- | --- | --- | --- | --- |
| 12a | Do you feel guilty because you have (had) leprosy?  *Waktu […] kena kusta, apakah […] rasa bersalah untuk keluarga/masyarakat ?* | 0 |  | 0 | 0 |  |  |  | |
| b | (If yes) How often has this happened?  *Kalo iya, sebarapa sering akang terjadi par [..]?* |  |  |  |  | 2 | 1 |  | |
| 13a | Do you feel you are not as good a person as others because you have (had) leprosy?  *Waktu […] kena kusta, apakah […] rasa seng sebaik orang lain dalam keluarga/masyarakat ?* | 0 |  | 0 | 0 |  |  |  | |
| b | (If yes) How often has this happened?  *Kalo iya, sebarapa sering akang terjadi par [..]?* |  |  |  |  | 2 | 1 |  | |
| 14a | Are you embarrassed that you have (had) leprosy?  *Waktu […] kena kusta, apakah […] rasa malu ?* | 0 |  | 0 | 0 |  |  |  | |
| b | *(If yes) How often has this happened?*  *Kalo iya, sebarapa sering akang terjadi par [..]?* |  |  |  |  | 2 | 1 |  | |
| 15a | Does having (had) leprosy make you feel unclean?  *Waktu […] kena kusta, apakah […] rasa diri seng barsih ?* | 0 |  | 0 | 0 |  |  |  | |
| b | (If yes) How often has this happened?  *Kalo iya, sebarapa sering akang terjadi par [..]?* |  |  |  |  | 2 | 1 |  | |
| 16a | Do you regret having told some people that you have (had) leprosy?  *Apakah [..]rasa manyasal su carita par beberapa orang kalo [..]pernah atau sementara kusta?* | 0 |  | 0 | 0 |  |  |  | |
| b | (If yes) How often has this happened?  *Kalo iya, sebarapa sering akang terjadi par [..]?* |  |  |  |  | 2 | 1 |  | |
| 17a | Does having (had) leprosy make you feel that you are a bad person?  *Waktu […] kena kusta, apakah […] rasa jadi bukang orang yang baik di keluarga/masyarakat ?* | 0 |  | 0 | 0 |  |  |  | |
| b | (If yes) How often has this happened?  *Kalo iya, sebarapa sering akang terjadi par [..]?* |  |  |  |  | 2 | 1 |  | |
|  |  | ***Sub total*** | | | | | | |  |

| **Anticipated stigma**  *We will read several statements/sentences […] please provide feedback, whether […] agree/disagree  **Kami akan membacakan beberapa pernyataan/kalimat […] mohon memberikan tanggapan, apakah […] setuju/tidak setuju* | | | | | | | | | |
| --- | --- | --- | --- | --- | --- | --- | --- | --- | --- |
| 18a | Do people affected by leprosy lose their jobs when their employers find out?  *Orang yang kana kusta seng bisa punya pekerjaan lai pas dong bos tau.*  *Apakah […] setuju dengan kalimat diatas ?*  **NB : Bila setuju, lingkari ya. Bila tidak, lingkari tidak* | 0 |  | 0 | 0 |  |  |  | |
| b | (If yes) How often does this happen?  *Kalo iya, sebarapa sering akang terjadi par [..]?* |  |  |  |  | 2 | 1 |  | |
| 19a | Are people affected by leprosy treated like a public nuisance?  *Orang yang kana kusta dapa biking kaya pengganggu di masyarakat*  *Apakah […] setuju dengan kalimat diatas ?*  **NB : Bila setuju, lingkari ya. Bila tidak, lingkari tidak* | 0 |  | 0 | 0 |  |  |  | |
| b | *(*If yes) How often does this happen?  *Kalo iya, sebarapa sering akang terjadi par [..]?* |  |  |  |  | 2 | 1 |  | |
| 20a | Do most people think that a person affected by leprosy is disgusting?  *Orang yang kana kusta itu biking gali (menjijikan)*  *Apakah […] setuju dengan kalimat diatas ?*  **NB : Bila setuju, lingkari ya. Bila tidak, lingkari tidak* | 0 |  | 0 | 0 |  |  |  | |
| b | (If yes) How often does this happen?  *Kalo iya, sebarapa sering akang terjadi par [..]?* |  |  |  |  | 2 | 1 |  | |
| 21a | Do most people feel uncomfortable around someone affected by leprosy?  *Orang rasa seng nyaman dekat-dekat orang-orang yang kana kusta*  *Apakah […] setuju dengan kalimat diatas ?*  **NB : Bila setuju, lingkari ya. Bila tidak, lingkari tidak* | 0 |  | 0 | 0 |  |  |  | |
| b | (If yes) How often does this happen?  *Kalo iya, sebarapa sering akang terjadi par [..]?* |  |  |  |  | 2 | 1 |  | |
|  |  | ***Sub total*** | | | | | | |  |
|  |  | ***Total*** | | | | | | |  |

Note: (normal words): English language; (*italic words*): Ambonese-Malay language

*The SARI Stigma Scale based on the Berger HIV stigma scale
